# Understanding Telerehabilitation Factors and Videoconference Usage in Physiotherapy: A Protocol for a Mixed‐Methods Project

**DOI:** 10.1002/hsr2.70287

**Published:** 2024-12-18

**Authors:** Nuria Gómez‐Molina, Iosune Salinas‐Bueno, Cristina Moreno‐Mulet

**Affiliations:** ^1^ Department of Nursing and Physiotherapy University of the Balearic Islands Palma Illes Balears Spain; ^2^ Health Research Institute of the Balearic Islands (IdISBa) Palma Spain

**Keywords:** focus groups, mixed methods, physiotherapy, semi‐structured interview, study design, survey, telerehabilitation, videoconferencing

## Abstract

**Background and Aims:**

The rapid evolution of healthcare technology introduced telerehabilitation (TR) as a novel intervention model. TR employs information and communication technologies for remote healthcare delivery. The COVID‐19 pandemic prompted a significant increase in TR usage, notably videoconferencing, among physiotherapists in Spain, offering a safe and viable alternative during mobility restrictions and temporary closure of physiotherapy centers. The aim of this study is to collect data on the use and frequency of outpatient TR and its intended purposes among physiotherapists from the pre‐COVID‐19 era to the present, as well as to examine the perspectives, facilitators, and barriers related to videoconferencing among physiotherapists, patients, and managers.

**Methods:**

A convergent two‐phase mixed‐methods project was designed. The first phase employs a quantitative methodology to explore how the frequency and purpose of physiotherapist TR usage changed from before the pandemic until the present, where 90% works at private practice and 10% at public services, using a retrospective online survey. Second, we focus on understanding videoconference usage through qualitative methodologies, which include focus groups with patients and physiotherapists and semi‐structured interviews with managers.

**Results:**

The expected results will be adoption and cessation of technology/ICT usage, changes in frequency and purpose of use; acceptance, satisfaction, and future expectations of videoconferencing by physiotherapists, patients, and managers.

**Conclusions:**

This project can be used to investigate how different telemedicine technologies have progressed in a region over a period and why healthcare professionals, patients, and managers embrace these technologies. As a result of this research, it will be possible to improve the implementation of these technologies, as well as professional training and educational programs.

## 1 Introduction

In the Balearic Islands (Spain) after the WHO declared the global COVID‐19 pandemic, the Government imposed containment policies, mainly lockdown and interpersonal contact limitations to prevent the spread of COVID‐19 from March 14, 2020, to June 21, 2020. Specific containment policies and their removal were regulated according to each regional healthcare centers' overload of COVID‐19 patients. In addition, healthcare centers generated distance policies, suspended non‐acute care activities, such as physiotherapy and allocated professionals to manage COVID‐19 [1, 2, 3]. Additionally, due to the increased number of critical COVID‐19 patients, multiple locations, such as physiotherapist wards, were used to place COVID‐19 patients [1]. In contrast, regarding private healthcare practice, where 90% of Spanish physiotherapists worked, were allowed to close physiotherapy centers except for the management of urgent pathologies [4]. Consequently, 90% of physiotherapy services were paralyzed for 1–3 months [3, 5]. As a result, telerehabilitation was a feasible and safe method for patients and physiotherapists to continue remote rehabilitation treatments despite containment policies [6, 7, 8, 9].

Telerehabilitation is a digital approach which includes the use of information and communication technologies (ICTs), where distance is an essential factor for the exchange of information, for examination, treatment, and prevention of pathologies [10, 11]. Telerehabilitation, whether with synchronous tools (phone calling or videoconferencing [VC]) or asynchronous (text messaging or electronic mailing) increased internationally during the pandemic health restrictions [12, 13]. This high usage creates the question of whether it was a technology that was here to stay, given the features it offered [14, 15].

The use of remote technologies involves different devices and serves various functions, especially in areas with high geographical dispersion [16]. Therefore, we will focus on VC because it provides synchronous visual and audio information for communication and promotes accurate interventions [17]. Moreover, VC research focused on feasibility, specific pathology protocols, and pretest interventions, focusing on the perceptions of professionals and patients [2, 18, 19, 20]. Consequently, VC was proven feasible and as effective as in‐person treatments for patients with musculoskeletal, neurological, chronic pathologies and seniors, as well as showing patient satisfaction [7, 21, 22, 23, 24, 25, 26]. In addition, VC is accessible, cost‐effective, safe, and provides patient contextualization [8, 17, 27].

However, research on the global view of VC as a tool, rather than as a specific pathology intervention using VC, is needed. Despite physiotherapists and patients had positive experiences using VC, how VC fits physiotherapists and patients' purposes after removing containment measures remain unclear [5, 6, 17]. As a result, it is currently unknown which TR technologies are still in use and why the ICTs have been abandoned after the pandemic, even though research supports their use. To promote TR adoption, it is necessary to understand the current use of TR and how it is perceived by professionals and patients.

The study aims to explore the use of telerehabilitation from the pre‐COVID‐19 era to the present and to determine perspectives, facilitators, and barriers to VC among physiotherapists, patients, and managers.

The specific objectives proposed are

− To explore the evolution of the use of ICT in telerehabilitation before, during, and after the first state of emergency in the Balearic Islands.
− To assess the impact of the pandemic on the clinical work of physiotherapists and the treatments of patients in the Balearic Islands.
− To understand the relationship between the lockdown and the increased use of ICT in telerehabilitation.
− To improve the understanding of the factors that act as facilitators and barriers to the use of VC in the practice of physiotherapists in the Balearic Islands.
− To assess the satisfaction and expectations of physiotherapists, patients, and managers regarding the use of VC in telerehabilitation.

## 2 Methods

### 2.1 Study Design

This is a convergent mixed‐methods project designed from a pragmatic paradigm composed of two different methodological parts as shown in the QUAN + QUAL project diagram (Annex S1). The quantitative phase, we will conduct a descriptive study to explore changes in physiotherapist TR use and purpose using a retrospective online survey. The qualitative phase, with an interpretative approach, we will focus on understanding the enabler and barrier factors related to VC usage by focus groups (FGs) with physiotherapists and patients, and interviews with managers.

For this project, the Balearic Islands region was selected because COVID‐19 containment measures were applied in the same way for the whole territory.

### 2.2 Quantitative Phase

This project will apply a retrospective online survey to map the change in TR use by physiotherapists before the pandemic until the present in the Balearic Islands.

As we had no record of a specific questionnaire for physiotherapists during the pandemic, we designed and pretest the survey with LimeSurvey (Version 3.25.9+210125) (Annex S2).

We conducted a pretest survey with 25 physiotherapists in the sample. The pretest gave us feedback to make focused questions including specific addressing information and to determine the time to complete the survey which ranged from 5 to 7 min.

#### 2.2.1 Sample Size and Participants

The target population is physiotherapists working in clinical settings in the Balearic Islands. Balearic physiotherapists will be contacted by the Regional Professional Association of Physiotherapists (RPAP). The target population comprises 1600 physiotherapists, so a sample size of 180 participants, with a confidence value of 95%, would be sufficient to determine the percentage of synchronous TR use as the principal variable with a precision of ±20% during the Spanish State of Emergency declared in March 2020, which is a population percentage that will predictably be around 50%. However, the use of an online survey involves self‐selection or voluntary sampling. Consequently, to ensure the availability of sufficient data for analysis, participation will be encouraged to achieve at least a 12% response rate from the study population.

#### 2.2.2 Data Collection

RPAP will send the survey link via email, the main communication method of the organization, to all physiotherapists in the Balearic Islands. To encourage the response rate, survey link will be sent via email to collaborators from the University of the Balearic Islands, and it will be shared on social media and instant messaging services. Moreover, the RPAP will send two reminders, and participation will be promoted through key informants from the participating centers.

The inclusion criteria for survey participants are

- Being a member of the RPAP.
- Having worked in the healthcare field at any time from January 1, 2020, until the survey's closure in the Balearic Islands.

To avoid response duplication, each physiotherapist must create a personal code using the first letters of both surnames and day of birth.

Participants will receive information sheet advice at the beginning of the survey to voluntarily engage in the project. Data collected from the survey will be anonymous, with a commitment to non‐identification.

##### 2.2.2.1 Sociodemographic Data

The survey will collect data on age, sex, professional data such as years of experience, work setting, main clinical specialty, and whether they have multiple jobs.

##### 2.2.2.2 Telerehabilitation Use

The survey consists of frequency and purpose questions from each ICT for periods. Periods are before the State of Emergency caused by COVID‐19 as T1, during the Spanish State of Emergency as T2 (March 14, 2020, to June 21, 2020), and when professionals will fill in the survey named T3.

Participants will be asked to answer per job they had whether they were working during each period to repeat questions related to TR usage.

##### 2.2.2.3 Frequency of Telerehabilitation Use

We classified the most used TR tools in physiotherapy from the literature review as phone calls, mobile applications or web platforms, electronic mail, videoconference, and instant messaging services. To report the frequency of use of each tool, a five‐point Likert scale will be used in the survey as an ordinal categorical variable answer option: *never, occasionally, once a week, 2–3 times per week, or daily*.

##### 2.2.2.4 Telerehabilitation Use Purpose

To report the primary purpose of each TR tool (phone calls, mobile applications, or web platforms, electronic mail, videoconference, and instant messaging services), participants will choose an answer from the following options: *I did not use it, assessment, treatment, monitoring, and sending information*.

##### 2.2.2.5 Workload Changes during the State of Emergency

We will measure the changes in the survey workload during T2 as to what extent do you agree with the following statements: *Your care work was affected, your care work was paralyzed, you performed support functions for other professionals, you treated patients with COVID‐19*, and *increased your assistance activity* using an agreement 5‐point Likert scale.

##### 2.2.2.6 Workload Changes Between the State of Emergency and When Professionals Will Fill in the Survey

Participants will be asked about changes in work burden and type of work during T3 with a dichotomous yes/no question: Have you returned to the same type and amount of work as before COVID‐19? If the subjects will select *No*, they will be able to explain why in an open clause.

Physiotherapists will be informed of the second part of the study at the end of the survey, where a dissociated survey link is shared. This link will provide a dissociated second survey which also filters the inclusion/exclusion criteria and will collect contact information about the work setting and VC experience.

#### 2.2.3 Data Analysis

We will perform descriptive statistics for the sample distribution, exposing medians of use frequency for each technology at T1, T2, and T3 as box plots for the frequency and bar charts for purpose of use of each technology. In addition, we will assess TR usage according to age, sex, years of experience, work setting, and principal specialism.

We will perform inferential statistical analysis using nonparametric tests due to their suitability, as the dependent study variables are ordinal and nominal categorical variables.

We hypothesized that the frequency of VC usage by physiotherapists at T1 would be different from that at T2 and T3. To analyze the change in TR frequency and purpose of use along T1, T2, and T3 for each participant, we will use IBM SPSS Statistics V.28 or the Friedman test to repeated measures analysis as a for statistical data analysis, with a *p*‐value < 0.05 will be considered significant. We also contrast the next hypotheses: (a) we propose that the number of physiotherapists who used VC increased at T2 from T1, (b) the number of physiotherapists who used VC decreased at T3 from T2, and (c) the number of physiotherapists who employed VC increased at T3 from T1. To analyze the increasing and decreasing use along T1, T2, and T3, we will dichotomize results as 0 related to the answer *never* and 1 to use, which include *occasionally, once a week, 2–3 times per week, or daily*.

We will perform McNemar's test, which is designed to analyze paired data with a *p*‐value < 0.05 will be considered significant. In addition, we will contrast secondary hypotheses using the Cochran‐*Q* test as a nonparametric test on paired nominal data.

Stratified data analysis will be performed using the Kruskal–Wallis test as a nonparametric comparison of the means of more than two groups for sex, age, years of experience, work settings, and Mann–Whitney *U* test for clinical specialisms.

### 2.3 Qualitative Phase

In the second part of the project, we will use a qualitative approach to understand the perspectives, experiences, and beliefs regarding using VC in physiotherapy. We employ an interpretative paradigm supported by the Technology Acceptance Model 3 (TAM3) framework [28, 29].

TAM3 has been proven to be a reliable model for predicting behavioral intention to use (BIU) for different technologies in work contexts [29]. TAM3 is based on Perceived Usefulness (PU) and Perceived Ease of Use (PEU) of attitudes towards using technology, BIU, and technology usage. According to TAM, PU is strongly related to BIU [28]. Moreover, TAM3 includes the Determinants of PU that relate to social influence processes (subjective norms, voluntariness, image, and social influences) and cognitive instrumental processes managed by the experience of use and voluntariness [30]. It also adds a Theoretical Model of the Determinants of PEU which shows that without specific technical knowledge, people use anchors (self‐efficacy, facilitating conditions, intrinsic motivation, computer playfulness, and emotions) in the process of deciding related to technologies, yet some adjustments influence PEU and explain how determinants influence BIU [31]. As a result, TAM3 properly assesses VC usage related to work and elsewhere as a model for a strength‐based framework guide to elucidate the types of factors and how these factors are related to BIU [30]. Additionally, the TAM3 framework is feasible for a wide range of people, including those with and without technological experience.

We selected FGs among peers for physiotherapists and patients to obtain knowledge, perspectives, and attitudes towards VC use in physiotherapy. In contrast, we chose individual semi‐structured interviews (SIs) as our approach with managers to avoid raising ethical issues as nonpublic discourses may emerge.

#### 2.3.1 Sampling Methods and Participants

Theoretical and purposive sampling will be used to obtain diverse experiences and opinions.

The collaborating centers for patients' recruitment for FG will be hospitals and health centers from the Balearic Public Health System, private hospitals, multidisciplinary rehabilitation centers, and physiotherapy centers. Each center will have at least one collaborating physiotherapist who will seek patients based on the eligibility criteria. Collaborators will inform the patients of the project and request their participation by phone call. After obtaining consent to participate, they will send their contact information (name, phone number, and electronic mail address) to the IP, who contact participants via telephone to arrange for the FGs. Table 1 explains the inclusion criteria for the FG and SI participants.

**Table 1:** Participant eligibility criteria for focus groups and semi‐structured interviews.

Patients	Undergoing PT treatment between March 1, 2020, to March 14, 2020.
	Balearic resident from T1 to T3.
	Have oratorical skills to discuss.
	Agree to participate in the project and sign the informed consent
Physiotherapists	Be a member of the Professional Association of Physiotherapists in the Balearic Islands.
	Have worked in a clinical setting at the Balearic Islands before Spanish Estate Alarm.
	Work in a clinical setting at the Balearic Islands at sample time.
	Agree to participate in the project and sign the informed consent.
Managers	Be a qualified healthcare professional
	Dedicate more than 50% of their working day to management tasks
	Carry out management tasks before the state of alarm on March 14, 2020
	Agree to participate in the project and sign the informed consent

Physiotherapist recruitment will be done via the second survey, designed for this purpose, by formal recruitment via RPAP email and informal through telephone, social media, and face‐to‐face contact. Additionally, snowball recruitment will be employed through participants. The recruitment of managers will be done sending an invitation via email and phone call.

FG's participants will be physiotherapists, patients, who share their experiences, testimonies, and opinions. Patients' FGs will be composed of patients, caregivers of cognitively disabled patients, or legal representatives of minors.

We established a minimum of three FG of physiotherapists, three FG of patients, and eight managers' SIs segmented as exposed on Table 2. In addition, the number of approaches will be determined by data saturation. FG participants will be segmented by public and concerted healthcare centers or private centers, wherever they will be patients or physiotherapists, because of that it will be at least one group of physiotherapists and one of patients both from public centers or concerted, and one physiotherapist FG and one patient FGs from private centers.

**Table 2:** Qualitative participant's segmentation.

Participants	Primary care	Public hospitals	Concerted hospitals	Private hospitals	Multidisciplinary centers	Physiotherapy center	Others	
Physiotherapists	1	3	3	3	1	3	3	17
Patients	1	3	3	3	3	3	3	19
Managers	1	2	1	1	1	1	1	8
Summation	3	8	7	7	5	7	7	44

#### 2.3.2 Data Collection

Before starting FG, each participant will receive the participant information sheet and will be able to solve any doubts. They will fill in an application form with the type of center which is physiotherapist, patient or manager, age, sex, and VC experience, to ensure FG segmentation. We will explain that participants can leave the project at any time without explaining and without consequence to their relationship with the researchers or healthcare professionals. Participants will voluntarily engage, and identifiable data will be coded to ensure confidentiality. We commit to updating the participants about project outcomes.

N.G‐.M. will perform FGs and SIs, with the support of C.M‐.M. and I.S‐.B. N.G‐.M. is an experienced healthcare female physiotherapist with an MSc in health research and high‐prevalence diseases, a PhD student, and a lecturer at the University of the Balearic Islands. Moreover, the IP reflected on the profession and her relationships with colleagues before approaching the sample, so as not to affect participants' positions.

The FG will consist of six to eight participants and last for 60–90 min. We will conduct SI and FG in persons at the University of the Balearic Islands' facilities, collaborator centers, or by VC, which is feasible, and the results will be comparable to in‐person [32]. Each participant takes part once in a FG or a SI.

The first SI and FG with patients and the one with physiotherapists will be pilot‐tested to ensure participant understanding. The proposed questions refer solely to participants' experiences (Annex S3).

FG and SI will be recorded via audio, and IP will take literal transcriptions with field notes after the sessions. Transcriptions will not include personal data or references to identify participants. To maintain the confidentiality of FG participants, we will assign a pseudonym to each participant using categorizing codes as P to patient, PT to physiotherapist, M to manager, adding a number related to participation order and VC if participants were experienced at VC related to physiotherapy treatment. We will not return transcripts to the FG participants to protect their data; instead, we will provide SI transcriptions to participants for their comments and corrections.

We will continue collecting information until data saturation, when no new themes emerge in subsequent FGs and SIs and following Malterud Siersma, and Guassora's [33] recommendations and the informative power of the participants will be achieved, to ensure validity and methodological accuracy.

#### 2.3.3 Data Analysis

We will perform a literal transcription of the recordings. Coding identification and categorization will be performed by inductive‐deductive thematic analysis supported by the TAM3 framework [29]. We will discuss these findings and data saturation between an experienced qualitative researcher, C.M‐.M.; a digital health experienced researcher, I.S‐.B.; and a PhD student, N.G‐.M. We will perform the coding tree, describe the major themes, and explain the discussion of these minor themes.

If new themes emerge from the data analysis, we will conduct a specific second‐stage sampling to improve analytical sophistication.

Moreover, we will present quotations from the participants to illustrate emerging themes.

Qualitative data will be managed using MAXQDA 2022 program.

We will triangulate and discuss qualitative outcomes from patients, physiotherapists, and managers, to better understand their intention to VC use in physiotherapy.

Table 3 sets out the operational objectives, specific objectives, and objective‐related issues for each data collection technique.

**Table 3:** Operational objectives, specific objectives, and objective‐related issues for each data collection technique.

Operational objectives	Specific objectives	Survey questions	Focus group and interview questions
1. To explore ICT use evolution in TR before, during, and after the State of Alarm in the Balearic Islands.	1.1. To explore and quantify the use of various modalities of TR by physiotherapists working in the healthcare sector before (T1), during (T2), and after the first state of alarm (T3) in the Balearic Islands.	How often did you use the following technologies to directly deal with patients (T1, T2, T2)?	PT: How do you use telerehabilitation technology?
		For what primary purpose did you use the following technologies to directly deal with patients?	PT: How has the use of these telerehabilitation technologies changed since the Pandemic?
	1.2. To analyze the sociodemographic and work‐related characteristics of professionals and their use of various TR modalities.	What setting do you perform, or have you performed your job in?	PT: How do you use telerehabilitation technology? Why?
2. To understand the impact of the pandemic on the healthcare work of physiotherapists and the treatment of patients in the Balearic Islands.	2.1. To understand how the pandemic affected the healthcare activities of physiotherapists and patient interventions during the lockdown caused by the declaration of the pandemic.	Were you working as a physiotherapist in care settings from March 15, 2020, to June 21, 2020?	PT: How did you experience the declaration of the state of alarm caused by the Pandemic? PT: How did the state of alarm affect your work? PT: How did you continue your healthcare work? M: How did the pandemic, especially the state of alarm, affect the unit's healthcare work? M: How was the care work managed during confinement?
	2.2. To analyze the work impact across different types of centers and specialties	Which setting do you perform or have you performed your primary job in? Which branches of physiotherapy do you practice?	
3. To analyze the relationship between the lockdown and the use of ICT in TR.	3.1. To examine the relationship between the lockdown period and the use of ICT in physiotherapy.	DURING THE CONFINEMENT from March 15, 2020, to June 21, 2020, how often did you use the following technologies to deal directly with patients?	How has the use of these telerehabilitation technologies changed since the Pandemic?
		How much you agree or disagree: − Your healthcare work was affected. − Your healthcare work was paralyzed. − You performed support functions for other professionals. − You treated COVID patients − Your assistance activity increased.	PT: How did you continue your healthcare work?
	3.2. To understand how the use of ICT in physiotherapy changed during the lockdown.	DURING THE CONFINEMENT from March 15, 2020, to June 21, 2020, For what primary purpose did you use the following technologies to directly deal with patients?	PT: How did you continue your healthcare work?
			PT: How did you experience the declaration of the state of alarm caused by the Pandemic? PT: How did the state of alarm affect your work?
4. To improve the understanding of factors acting as facilitators and barriers to the use of VC in physiotherapy practice in the Balearic Islands	4.1. To analyze the sociodemographic and work‐related characteristics of professionals and their use of various TR modalities.	Which branches of physiotherapy do you practice? How often did you use the following technologies to directly deal with patients?	
	4.2. To explore the factors that act as facilitators and barriers to the use of VC in TR by physiotherapists, patients, and managers.		PT and M: How has the use of these telerehabilitation technologies changed since the Pandemic?
5. To understand the satisfaction and expectations of physiotherapists, patients, and managers regarding the use of VC in TR.	To explore the beliefs and perceptions of patients, professionals, and managers regarding the use of VC in physiotherapy.		P: How did you communicate with your physiotherapist? How did this communication proceed?
	To interpret the perceived future potential possibilities of VC by physiotherapists, patients, and managers.		M: What telerehabilitation technologies do you plan to implement in the unit?

Abbreviations: M, managers; P, patients; PT, physiotherapists.

### 2.4 Quantitative and Qualitative Data Integration

Once the qualitative and quantitative data will have been collected and analyzed, we will proceed to identify common themes between the two sets of results. Subsequently, a detailed analysis will be undertaken to determine how the qualitative and quantitative results will either confirm, expand or contradict each other. Confirmation will occur when the results of both data types will support one another, thereby enhancing the overall credibility [34]. Expansion will take place when the results from both data sources diverge and provide a broader understanding of the phenomenon under study by highlighting complementary aspects of the central topic. Conversely, contradiction will arise when the qualitative and quantitative data are inconsistent, incongruent, or contradictory [35]. This approach will enable an evaluation of the coherence or dissonance between the quantitative and qualitative findings, and how these findings will collectively offer a thorough and comprehensive understanding of the investigated phenomenon, thus addressing the research objectives [36].

The results of the survey, the FG, and the SI tend to improve the understanding of specific themes.

In addition, we will discuss the results of previous research to enhance the accuracy.

In Table 4, the project execution timeline is exposed.

**Table 4:** Project execution timeline.

	Data collection	Data analysis	Integration
Tasks			
To ask RPAP for collaboration sending the survey link	Survey collection	Data depuration Statistic analysis	To identify common themes To determine how data confirm, expand, or contradict each other
Qualitative physiotherapist and manager recruitment Find collaborators to patient recruitment.	Focus groups Semi‐structured interviews	Data transcription Thematic analysis	
Expected period			
1–6 months	1–6 months	3–6 months	1 month

### 2.5 Data Management

All participants will receive a participant information sheet containing all the information about the study and their voluntary participation in a form tailored to patients and professionals. In addition, they will be able to solve any questions related to their participation before the FG or SI. Moreover, all participants must sign the informed consent. Informed consent state that participants have been adequately informed, they agreed with their participation, and they can revoke their consent at any time without any prejudice. FG participants will also commit to protect others identifiable participants data.

All researchers will sign a confidence commitment to protect identifiable participant's data. Survey will collect anonymous data and researchers will be committed not to reidentify participants. Related to qualitative participant's data, they will be pseudonymised at transcription process. Only the IP, who will handle transcriptions, will know which pseudonym were related to each participant to ensure also that participants could revoke their participation consent. FG and SI recordings will be saved in a password‐protected folder.

Participant's contact data will be stored for 5 years on a registered fold of the University of the Balearic Islands in accordance with current Spanish legislation.

This Project was approved by the Research Ethics Committee of the Balearic Islands (CEI‐IB) with the code number IB 4928/22 PI.

## 3 Expected Results

Outcomes will expose which ICT best fitted among physiotherapist, explaining which frequency and purpose of use and how it changed along the period and how the pandemic period affected its adoption.

In addition, results integration will clarify how each ICT were more appropriate in a type of setting, clinical specialty or attended population.

Moreover, qualitative results will explain VC acceptance between patients and professionals, which barriers and facilitators, as patient with long‐term conditions, professional training and planned implementation, affected to its adoption and which use will be expected.

## 4 Discussion and Implications

The main purpose of this study is to understand the evolution of TR and VC use in the Balearic Islands over 3 years, to determine which factors act as enablers or barriers and to what extent they affect their choice of VC or other TR technology use. As a result, it will be known why VC was used and which technology was more appropriate for physiotherapists at different settings and users.

Moreover, research outcomes will be compared internationally and discussed to determine which would be major factors that may be affecting ICT adoption and whether they would be transferable to other regions.

The outcomes will provide evidence of how the COVID‐19 pandemic, when distance policies were mandatory, affected TR frequency and purpose use among physiotherapists. Otherwise, this project offers a research methodology and survey that can be adapted to other locations and periods.

The findings from this research will provide insights into best practices and areas needing improvement, as professional training, which can inform the implementation of TR technologies and the development of targeted physiotherapist educational programs.

This project will report on the situation and the need to develop health strategies that include the adoption of technologies to facilitate patient access to physiotherapy care. Additionally, it highlights the necessity for the development of specific legislation that establishes the foundations and limits of remote care to ensure a consolidated and safe practice for both patients and professionals.

Future research would address long‐term technology cost‐effectiveness, individual, and community benefits and service sustainability.

## 5 Limitations

The authors acknowledge several limitations of this study.

As participation in this study will be voluntary, researchers anticipate that participants are more likely to be familiar with technology or have prior experience with telerehabilitation. Consequently, self‐selection bias is expected to be a limitation that may impact results generalizability.

The qualitative methodology, a component of this mixed methods protocol, is by nature local and contextual, requiring consideration of demographic and professional variables specific to physiotherapy within this region. However, the use of the TAM3 theoretical framework will enhance the transferability of findings related to technology use to other regions and contexts.

The FG approach was selected as it provides more discursive themes than SI. Nevertheless, FG participants arrangement could be difficult. In case of sample recruitment difficulties due to arrangement issues, we alternatively propose to conduct SIs to participants, which could include from one to three participants.

## Author Contributions

**Nuria Gómez‐Molina:** conceptualization, investigation, methodology, writing–original draft, writing–review and editing. **Iosune Salinas‐Bueno:** conceptualization, methodology, supervision, writing–review and editing. **Cristina Moreno‐Mulet:** conceptualization, methodology, supervision, writing–review and editing.

## Conflicts of Interest

The authors declare no conflicts of interest.

## Transparency Statement

The lead author Iosune Salinas‐Bueno affirms that this manuscript is an honest, accurate, and transparent account of the study being reported; that no important aspects of the study have been omitted; and that any discrepancies from the study as planned (and, if relevant, registered) have been explained.

## Supporting information

Supporting information.

Supporting information.

Supporting information.

## Data Availability

Raw data will be generated at Nuria Gómez. Derived data supporting the findings of this study are available from the corresponding author (I.S‐.B.) on request.
