# Supplementary material for: Understanding Telerehabilitation Factors and Videoconference Usage in Physiotherapy: A Protocol for a Mixed‐Methods Project
Source: Health Sci Rep. 2024 Dec 18;7(12):e70287. doi: 10.1002/hsr2.70287 (PMC11655917; doi:10.1002/hsr2.70287)
Supplement: Supplementary file 1 — Supporting information. [file HSR2-7-e70287-s002.pdf]

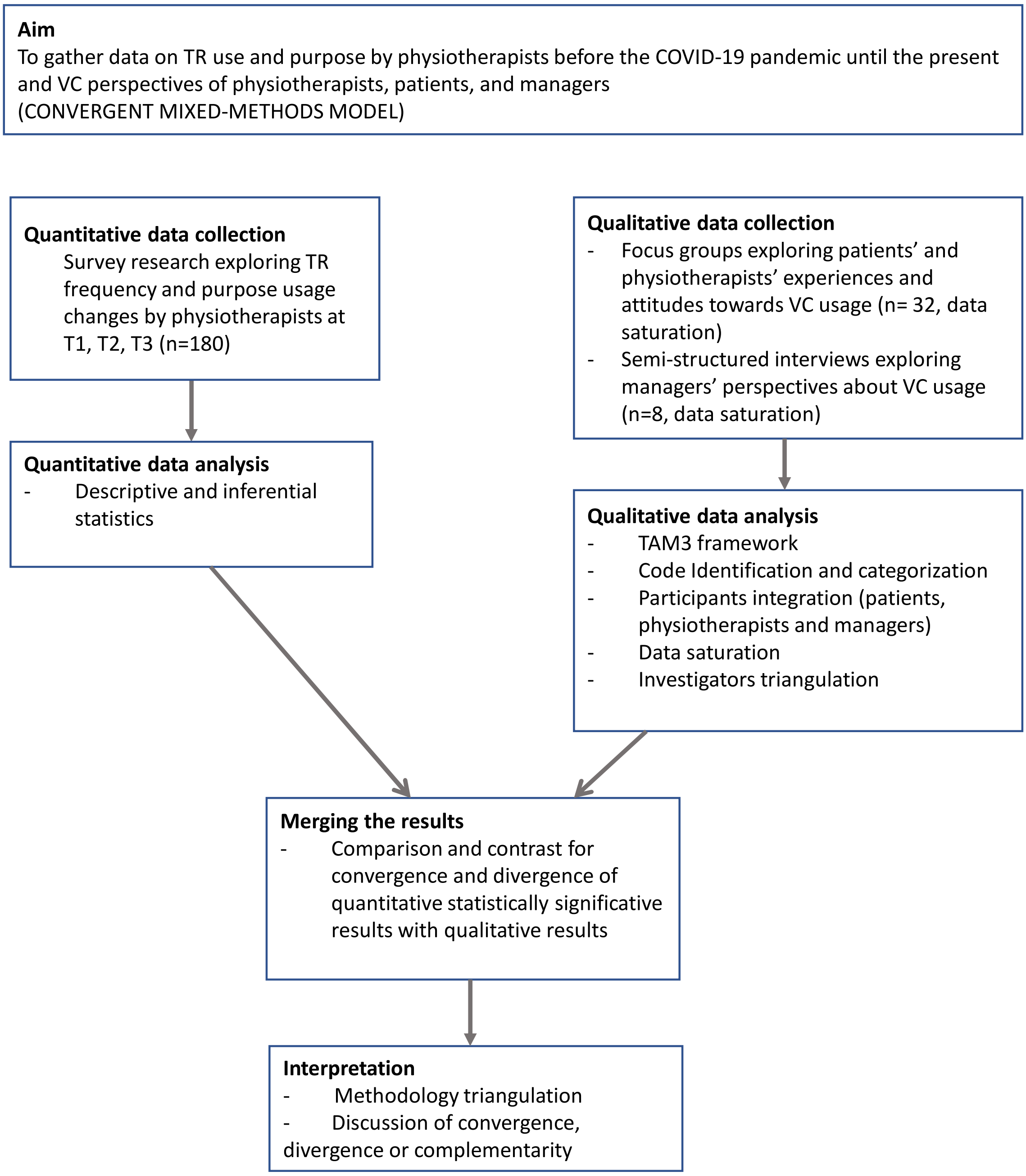
Aim
To gather data on TR use and purpose by physiotherapists before the COVID-19 pandemic until the present and VC perspectives of physiotherapists, patients, and managers
(CONVERGENT MIXED-METHODS MODEL)
Quantitative data collection
Survey research exploring TR frequency and purpose usage changes by physiotherapists at T1, T2, T3 (n=180)
Qualitative data collection
- Focus groups exploring patients' and physiotherapists' experiences and attitudes towards VC usage (n= 32, data saturation)
- Semi-structured interviews exploring managers' perspectives about VC usage (n=8, data saturation)
Quantitative data analysis
- Descriptive and inferential statistics
Qualitative data analysis
- TAM3 framework
- Code Identification and categorization
- Participants integration (patients, physiotherapists and managers)
- Data saturation
- Investigators triangulation
Merging the results
- Comparison and contrast for convergence and divergence of quantitative statistically significative results with qualitative results
Interpretation
- Methodology triangulation
- Discussion of convergence, divergence or complementarity
