# Supplementary material for: Understanding Telerehabilitation Factors and Videoconference Usage in Physiotherapy: A Protocol for a Mixed‐Methods Project
Source: Health Sci Rep. 2024 Dec 18;7(12):e70287. doi: 10.1002/hsr2.70287 (PMC11655917; doi:10.1002/hsr2.70287)
Supplement: Supplementary file 2 — Supporting information. [file HSR2-7-e70287-s001.docx]

Information and Communication Technologies for Physiotherapy in the Balearic Islands

**Description**

The aim of this questionnaire is to determine the use of information and communication technologies (ICT) for physiotherapy, as well as the changes that have occurred as a result of the pandemic generated by Covid-19 in the practice of physiotherapy in the Balearic Islands.

The first part consists of answering an online questionnaire addressed to physiotherapists residing and working in the Balearic Islands.

The questions concern the use of ICT in its various forms, before, during the confinement (March 14, 2020) and at present, as well as how the work activity was affected during the confinement.

**Introduction**

Your assistance is important. Answering this questionnaire will take less than 5 minutes. This questionnaire is part of a thesis project, and we need to reach as many physiotherapists as possible in the Balearic Islands. Complete and share this! Thank you very much, and may the strength be with you.

**Sociodemographic data**

The following questions are socio-demographic and labour-related. As for the questions related to jobs, they refer only to the period from January 2020 to the present.

1. Create a code with the initial of your first last name, the initial of your second last name, and the day number of your birth.

This code is necessary for the recovery of the questionnaire by the participant if he/she does not finish it and, at the same time, to avoid duplicate answers.

Example: If the first surname is Gómez and the second is Molina, and you were born on the 19th, your code would be GM19.

This code maintains the anonymity of the identity.

Please write your answer here:__________________________________

1. Have you worked as a care physiotherapist in the Balearic Islands since January 2020? Yes/No
2. Age: 21-70
3. Years of experience: 1-50
4. Which sector do you perform or have you performed your primary job in?* Please select only one of the following options

- Primary Care
- Public Hospital
- Concerted Hospital
- Private Hospital
- Residence or day centre
- Multidisciplinary centre
- Physiotherapy centre
- Physiotherapy at home
- Education centre
- Sport centre
- Other

1. Are you self-employed or employed?* Check the options that apply Self-employed/employed
2. On which Balearic Island do you live? Mallorca/Menorca/Ibiza/Formentera
3. Do you work or have you worked in more than one position simultaneously as a care physiotherapist from January 2020 to the present? Yes/No
4. In your main job, which branches of physiotherapy do you practice?*

Check the options that apply

- Traumatology and rheumatology
- Neurology
- Cardiovascular and respiratory
- Paediatrics
- Pelvic floor
- Sport
- Geriatrics

1. Were you working as a care physiotherapist before the declaration of the state of alarm on 14 March 2020? Yes/No

**Before the Covid-19 Pandemic**

|  | I did not use it | Assessment | treatment | Monitoring | Sending information |
| --- | --- | --- | --- | --- | --- |
| Phone call |  |  |  |  |  |
| Apps or web platforms |  |  |  |  |  |
| Electronic mail |  |  |  |  |  |
| Videconference |  |  |  |  |  |
| Instant messages services (SMS, whatsapp, telegram,…) |  |  |  |  |  |

1. BEFORE COVID-19 In your main job, for what primary purpose did you use the following technologies to directly deal with patients/users?*

This question refers to ONLY to the communication between physiotherapist and patient, and excludes the administrative process of making appointments.

1. BEFORE COVID-19 In your main job, how often did you use the following technologies to directly deal with patients?*

|  | Never | Occasionally | Once a week | 2-3 times per week | Daily |
| --- | --- | --- | --- | --- | --- |
| Phone calls |  |  |  |  |  |
| Apps or web platforms |  |  |  |  |  |
| Electronic mail |  |  |  |  |  |
| Videoconferences |  |  |  |  |  |
| Instant messages services (SMS, WhatsApp, telegram,…) |  |  |  |  |  |

This question refers to ONLY to the communication between physiotherapist and patient, and excludes the administrative process of making appointments.

**During the confinement caused by the Covid-19**

1. DURING THE CONFINEMENT from 03/15/2020 to 06/21/2020 In your main job. For what primary purpose did you use the following technologies to directly deal with patients/users?*

This question refers to ONLY to the communication between physiotherapist and patient, and excludes the administrative process of making appointments.

|  | I did not use it | Assessment | treatment | Monitoring | Sending information |
| --- | --- | --- | --- | --- | --- |
| Phone calls |  |  |  |  |  |
| Apps or web platforms |  |  |  |  |  |
| Electronic mail |  |  |  |  |  |
| Videoconferences |  |  |  |  |  |
| Instant messages services (SMS, WhatsApp, telegram,…) |  |  |  |  |  |

|  | Never | Occasionally | Once a week | 2-3 times per week | Daily |
| --- | --- | --- | --- | --- | --- |
| Phone calls |  |  |  |  |  |
| Apps or web platforms |  |  |  |  |  |
| Electronic mail |  |  |  |  |  |
| Videoconferences |  |  |  |  |  |
| Instant messages services (SMS, WhatsApp, telegram,…) |  |  |  |  |  |

1. DURING THE CONFINEMENT from 03/15/2020 to 06/21/2020 In your main job, how often did you use the following technologies to deal directly with patients? *

This question refers to ONLY to the communication between physiotherapist and patient, and excludes the administrative process of making appointments

1. In relation to the following statements in your main job select how much you agree or disagree with them:*

|  | Strongly agree | Agree | Neither agree nor disagree | Disagree | Strongly disagree |
| --- | --- | --- | --- | --- | --- |
| Your healthcare work was affected |  |  |  |  |  |
| Your healthcare work was paralyzed |  |  |  |  |  |
| You performed support functions for other professionals |  |  |  |  |  |
| You treated COVID patients |  |  |  |  |  |
| Increased your assistance activity |  |  |  |  |  |

**Current job**

1. Are you currently working as a physiotherapist in care settings? Yes/No*
2. Currently, in your main job, for what primary purpose do you use the following technologies to directly deal with patients/users?*

This question refers to ONLY to the communication between physiotherapist and patient, and excludes the administrative process of making appointments.

|  | I do not use it | Assessment | treatment | Monitoring | Sending information |
| --- | --- | --- | --- | --- | --- |
| Phone call |  |  |  |  |  |
| Apps or web platforms |  |  |  |  |  |
| Electronic mail |  |  |  |  |  |
| Videoconference |  |  |  |  |  |
| Instant messages services (SMS, WhatsApp, telegram,…) |  |  |  |  |  |

1. Currently, in your main job, how often do you use the following technologies to directly deal with patients?*

This question refers to ONLY to the communication between physiotherapist and patient, and excludes the administrative process of making appointments.

|  | Never | Occasionally | Once a week | 2-3 times per week | Daily |
| --- | --- | --- | --- | --- | --- |
| Phone calls |  |  |  |  |  |
| Apps or web platforms |  |  |  |  |  |
| Electronic mail |  |  |  |  |  |
| Videoconferences |  |  |  |  |  |
| Instant messages services (SMS, WhatsApp, telegram,…) |  |  |  |  |  |

1. Have you returned to the same type and amount of work as before Covid-19? Yes/No*
2. If your answer is no, how has it changed?*

Please write your answer here:_____________________________________________________________________

This project consists of a second part in which discussion groups will be held between 6-8 physiotherapists in order to learn more about the experiences and points of view on Telerehabilitation and, especially, videoconferencing in physiotherapy.

Your experience is important whether you use the video call or not, so we encourage you to participate.

If you wish to participate and obtain more information, please click on the following link.

**Link ⇒ Focus Group**

If you have made it this far, thank you. Thank you for your time and collaboration regarding this study

This helps physical therapy grow as a profession.

*

* These questions will be repeated wherever they answer “Yes” to Question 8, changing the sentence “in your main job” and/or adding “in your second job”
