# Supplementary material for: Understanding Telerehabilitation Factors and Videoconference Usage in Physiotherapy: A Protocol for a Mixed‐Methods Project
Source: Health Sci Rep. 2024 Dec 18;7(12):e70287. doi: 10.1002/hsr2.70287 (PMC11655917; doi:10.1002/hsr2.70287)
Supplement: Supplementary file 3 — Supporting information. [file HSR2-7-e70287-s003.docx]

**Annex 3. Focus groups and interview scripts**

**Focus groups script with patients**

What is your use of technologies such as WhatsApp, email, text messaging, health apps, and video calling?

What were you using before the pandemic?

How did your use change during confinement?

How do you feel when using these technologies?

You were undergoing physiotherapy when the State of Alarm was declared.

How did you live that moment? What did you do about your pathology? How did it evolve?

How did you feel about your treatment?

How did you communicate with your physical therapist? How did this communication proceed?

**Focus groups script with physiotherapists**

What use do you make of communication technologies on a personal level?

How do you use telerehabilitation technology?

How has the use of these telerehabilitation technologies changed since the Pandemic?

How did you experience the declaration of the state of alarm caused by the Pandemic?

How did the state of alarm affect your work?

How did you continue your healthcare work?

**Semi-structured interview script with managers**

What is the unit's current use of telerehabilitation technologies?

How has the use of these telerehabilitation technologies changed since the Pandemic?

How did the pandemic, especially the state of alarm, affect the unit's healthcare work?

How was the care work managed during confinement?

How can videoconferencing be implemented in your unit?

What telerehabilitation technologies do you plan to implement in the unit?
